# Documentation of in-hospital falls on incident reports: Qualitative investigation of an imperfect process

**DOI:** 10.1186/1472-6963-8-254

**Published:** 2008-12-11

**Authors:** Terry P Haines, Petrea Cornwell, Jennifer Fleming, Paul Varghese, Len Gray

**Affiliations:** 1 School of Primary Health Care, Department of Physiotherapy, Monash University, Frankston, Australia; 2 Continuing Care, Southern Health, Cheltenham, Australia; 3 School of Health and Rehabilitation Sciences, The University of Queensland, St Lucia, Australia; 4 Princess Alexandra Hospital, Woolloongabba, Australia; 5 Academic Unit in Geriatric Medicine, The University of Queensland, Woolloongabba, Australia

## Abstract

**Background:**

Incident reporting is the prevailing approach to gathering data on accidental falls in hospitals for both research and quality assurance purposes, though is of questionable quality as staff time pressures, perception of blame and other factors are thought to contribute to under-reporting.

**Methods:**

This research aimed to identify contextual factors influencing recording of in-hospital falls on incident reports. A qualitative multi-centre investigation using an open written response questionnaire was undertaken. Participants were asked to describe any factors that made them feel more or less likely to record a fall on an incident report. 212 hospital staff from 30 wards in 7 hospitals in Queensland, Australia provided a response. A framework approach was employed to identify and understand inter-relationships between emergent categories.

**Results:**

Three main categories were developed. The first, determinants of reporting, describes a hierarchical structure of primary (principle of reporting), secondary (patient injury), and tertiary determinants that influenced the likelihood that an in-hospital fall would be recorded on an incident report. The tertiary determinants frequently had an inconsistent effect. The second and third main categories described environmental/cultural facilitators and barriers respectively which form a background upon which the determinants of reporting exists.

**Conclusion:**

A distinctive framework with clear differences to recording of other types of adverse events on incident reports was apparent. Providing information to hospital staff regarding the purpose of incident reporting and the usefulness of incident reporting for preventing future falls may improve incident reporting practices.

## Background

Reporting of falls on hospital incident reports is an accepted standard for collating falls data in both clinical practice and research. [1-4] Concerns have previously been expressed regarding the ability of this system to accurately measure the "true" number of falls taking place on hospital wards.[3] In particular, discrepancies in the definition of a fall used in different facilities, time pressures on staff and the existence of a "blame" culture have been postulated to contribute to inconsistency in reporting and under-reporting respectively.[3,5,6]

It is plausible that more factors than these may be impacting upon the recording of in-hospital falls on incident reports. Although previous research has been conducted identifying barriers to incident reporting more generally, [6-8] specific investigation of reporting of in-hospital falls has not been undertaken. There are several differences between falls and other reportable adverse events that may give rise to a differing set of barriers and facilitators to their reporting. These include the immediate nature of falls (in comparison to other adverse events such as development of a pressure ulcer),[7] the high proportion that result in little physical injury,[1] and the relative contribution that patients and staff may play in their causality.[3]

We aim to identify contextual factors that influence the reporting of in-hospital falls on incident reports so that strategies to address barriers and maximize the value of facilitators can be devised.

## Methods

### Design

A multidisciplinary research team based at one of the participating sites commenced a quantitative and qualitative multi-centre investigation into in-hospital falls reporting using an open written response questionnaire. The intent of this study was to understand the contextual factors surrounding the consistency of falls incident reporting that would have been difficult to glean using quantitative techniques. It was envisaged that this would facilitate development of strategies to improve the completeness and consistency of falls reporting within and between hospitals.

### Setting and participants

#### Description of participating sites and participants

This multi-centre investigation was carried out across 30 wards in seven hospitals in Queensland, Australia. The investigators intended to sample hospitals across a range of geographical and funding system backgrounds to obtain a broad perspective on this issue. Facilities were recruited via electronic advertising in the Queensland Health Falls Injury Prevention Network. This resulted in recruitment of two publicly funded metropolitan teaching hospitals, one privately funded metropolitan teaching hospital, three publicly funded regional/rural hospitals, and one privately funded regional hospital. The investigative team (with a falls prevention research agenda) was based at one of the publicly funded metropolitan teaching hospitals. The investigators also intended to sample wards treating a range of patient diagnostic groups, for whom reported falls were highly prevalent. The wards sampled included geriatric rehabilitation, orthopaedic, neurological, general medical, and wards with mixed diagnostic groups. Nursing staff working over a pre-specified 24 hour period on the participating wards were asked to complete the questionnaire. A 24 hour selection was chosen so that proportional representation of day and night shift nursing staff could be sampled. Allied health staff (occupational therapy, physiotherapy) working on each ward were also targeted as they are also commonly involved in recording fall-related incident reports. Questionnaires were distributed in hard copy to staff by unit managers before or after "handover" and were returned to a survey collection envelope kept within their staff room by the end of the shift.

#### Process for completing incident reports

The process for completing incident reports at participating sites was via a computerized incident reporting system designed to capture all types of adverse events. This system primarily employed discrete item selection buttons with few open text fields. No uniform falls-reporting protocol was held across participating facilities at the time of investigation. A majority of participating sites had transitioned from a paper-based incident reporting system to a computer based system within the previous two years. National best-practice guidelines released just prior to project commencement specified that an incident report should be completed for all falls, regardless of where the fall occurred or whether the person was injured.[9] The process surrounding incident report completion for falls was the focus of this study however incident reports were not used as data themselves.

#### Measures

This investigation was conducted as a part of a broader survey of fall reporting practices in the hospital setting. An open-ended written, paper-based response question was asked of all participants. Limited demographic data was collected, specifically participant professional group, ward, hospital, and years of professional experience. This approach to data collection was selected as the investigators desired open and honest responses that could potentially have been critical of their employers, working conditions or that may have revealed deficiencies in their own professional practice. To glean this information, it was considered extremely important that anonymity and staff perception of anonymity be ensured through the study design. Face-to-face, or focus group interviews were therefore not selected on this basis. The question posed to participants was "Describe any factors that make you feel more or less likely to record a fall on an incident report", with an additional prompt of "what barriers do you face in completing incident reports for falls" also being provided. This prompt was included as prior clinical and research experiences of the investigative team had led them to hypothesize that there may be under-reporting of in-hospital falls on incident reports due to as yet unidentified barriers. The remainder of the survey focused on whether or not hospital staff classified several video scenarios as falls (data not presented in this manuscript).

#### Analytical methods

A framework approach was undertaken for the data analysis. This approach uses content analysis techniques and incorporates 5 stages of data analysis; i) familiarization with the raw data, ii) identifying a thematic framework that separates the data into manageable portions, iii) indexing by applying codes to the text, iv) charting the data to the appropriate part of the thematic framework to which they relate, and v) mapping and interpretation to define concepts and find associations between categories.[10] All responses were typed and compiled into a single Microsoft Word document. Responses were initially reviewed and separated into individual comments by the principal investigator (TH). This investigator used the emergent categories of "the act of reporting" and "reasons for reporting" to initially code individual comments. The reasons for reporting category encompassed comments describing the considerations that staff members took into account when deciding whether a patient had fallen or not and that influenced whether they thought an incident report should be completed. The act of reporting category encompassed comments describing practical issues that arose when trying to perform the act of completing an incident report. A second investigator (PC) then examined the initial coding, contested the labeling and allocation of comments to categories where deemed appropriate, and assisted with further data analysis and theorization. Where disagreements over coding and subsequent theorization could not be resolved, a third investigator (JF) arbitrated. The data were then re-examined and broken down into multiple smaller categories. These emergent categories were then examined for cross-relations by examining responses from respondents who provided multiple comments and responses where individual comments provided information across categories. This led to development of "main categories" labeled "environmental/cultural facilitators", "environmental/cultural barriers", and "determinants of reporting". Inter-relations between individual categories within and between these main categories were examined and graphically conceptualized. Finally, original data were again re-examined to determine if there were further emergent categories not yet described and integrated into the framework, to evaluate whether main category and category labels adequately described the content of comments contained within, and to ensure that comments originally placed in each category still belonged there. Quotations presented were selected on the basis of investigator (TH&PC) determination that they most aptly represented the substance of the category it was being used to describe. A definitive graphical representation of the relationships between categories within and between main categories was developed.

#### Ethics

Ethics committee approval was provided by local hospital ethics committees and The University of Queensland Medical Research Ethics Committee. Written informed consent was not sought from individual participants, rather written gatekeeper consent was gained from departmental managers and nurse unit managers, while implied consent was gained from respondents when submitting their survey. No personal checks were made to ensure that surveys were completed, staff were free to submit a blank survey form if they did not want to respond to the questions provided. Investigators TH, PC and JF held "research only" positions within one of the facilities where the survey took place and did not have a direct clinical role with patients or supervisory role with staff members who participated in the survey. PV and LG did have direct clinical roles with patient care and supervisory roles with staff members who participated in the study but were not involved in data analysis or interpretation.

## Results

Respondents were drawn from a range of hospital wards including geriatric rehabilitation (n = 153), general medical/surgical (n = 73), neurological (acute/rehabilitation) (n = 59), orthopaedic (n = 55), other wards (n = 106). A total of 446 survey response forms were submitted, incorporating nursing staff (n = 329, 73.8%), physiotherapists (n = 66, 14.8%), occupational therapists (n = 25, 5.6%), and other health professionals (n = 26, 5.8%). The mean (sd) number of years of professional experience of respondents was 11.8 (10.5) years. Of the 446 survey response forms submitted, 212 (48%) in total provided a written response to the open question posed for this research. There were 416 comments generated from the 212 responses. The greatest number of comments on any one survey response was five.

**Figure 1:**
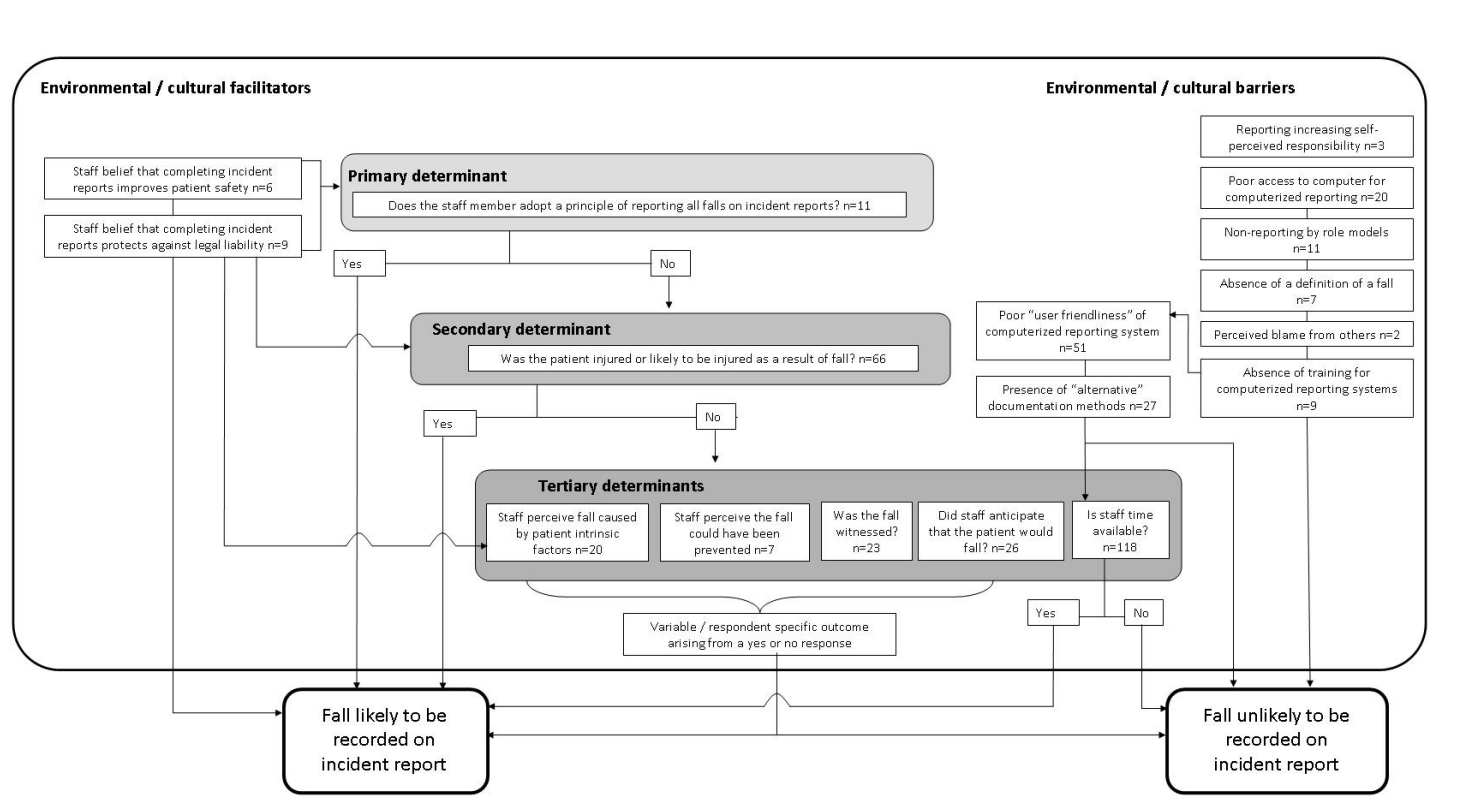
Conceptual diagram of factors likely to impact upon whether a fall is recorded on an incident report.

Analysis of the data led to the development of 17 categories which were divided into the main categories of "environmental/cultural facilitators" (two categories), "environmental/cultural barriers" (eight categories), and "determinants of reporting" (seven categories). Inter-relations between these main categories and categories between and within main categories are demonstrated (figure 1). The determinants of reporting main category contained within it a hierarchical structure of categories (primary, secondary and tertiary determinants) that relate to the circumstances and outcomes of the fall, the attitude to reporting held by the staff member, and how busy the staff member is at the time of the fall. These determinants exist upon a context of environmental and cultural factors that may act as barriers or facilitators to reporting falls on incident reports. Thus conceptually, there is a degree of inter-relationship between all the cultural/environmental factors with the determinants of reporting, though more specific links are demonstrated by arrows within the figure and are further discussed.

### Determinants of reporting – primary determinant

The primary determinant was whether a staff member adopts a principle of reporting all falls on incident reports. This principle was exemplified in the statements made by respondents;

"I would document any fall with an incident report to prevent the same mistake happening again and ensure the pt (patient) was as safe as possible under our duty of care as health professionals" (r)146, occupational therapist

"Every fall needs incident reports to be completed without or with injuries for both nurse and pt's protection for future references or if needed further investigations as required." r97, nurse.

Staff appeared to adopt this principle if they believed that completing incident reports for falls improved patient safety and/or protected them against legal liability. The links between the primary determinant and these "environmental/cultural facilitator" categories are demonstrated (figure 1). Only a small number of comments were received indicating that respondents adopted this principle however.

### Determinants of reporting – Secondary determinant

A larger number of comments (n = 66) were received indicating that staff would be more inclined to record an incident report if the patient were injured. For example;

"More likely to record a fall if the person was injured." r120, nurse

These comments imply that the respondents did not complete an incident report for all falls, particularly where a patient was not injured. The propensity for staff to record incident reports where a patient is injured may be motivated by a desire of staff to protect themselves from future litigation, as demonstrated by the following;

"More likely to report a fall if I was directly in contact...to cover against liability. Less likely to record incident report if person is clearly not hurt." r144, occupational therapist.

The link between the belief that completing an incident report protects against legal liability and the greater propensity to complete an incident report if the patient was injured is demonstrated in figure 1.

### Determinants of reporting – Tertiary determinants

The responses received also indicated there were a number of tertiary determinants, though their subservience to the presence or potential for patient injury in the hierarchical structure of this main category was frequently evident. The following example illustrates this;

"It would depend on the circumstances to the pt (patient) specifically. If a pt is injured I would always fill out an incident report. If it was just poor technique and the pt can and should have known and done better then I probably won't fill one out." r121, nurse

This demonstrates that the tertiary determinant "staff perceive the fall could have been prevented" would affect this respondent's reporting practices but is superseded by the secondary determinant "was the patient injured or likely to be injured as a result of fall".

Similarly the statement

"time factor for completing incident report – if pt not reporting any deficits especially" r139, occupational therapist

demonstrates the presence of the tertiary determinant "is staff time available", while also highlighting that it's influence is contingent upon whether the patient was injured.

The tertiary determinant "is staff time available" was most frequently referred to of all the tertiary determinants. It encapsulated both an overall workload dimension,

*"(the computer program) takes a long time to complete, unlike the previous paper based reports. If the ward is very busy, reporting is sometimes neglected." r119, nurse*, and a timing of the fall dimension, particularly related to falls that occur at the end of their shift. For example;

"When a minor fall may occur towards the end of a shift and there are other acute patient issues to manage. Difficult to expect staff to work overtime to complete incident report" r80, nurse.

The "is staff time available" tertiary determinant was related to the environmental/cultural factor of "poor user friendliness of computerized reporting system", as demonstrated by the statement by r119 above, and by the statement;

"When a minor fall may occur towards the end of a shift and there are other acute patient issues to manage. Difficult to expect staff to work overtime to complete incident report" r80, nurse.

The statement by r119 also related the "is staff time available" tertiary determinant to the environmental/cultural factor of "presence of alternative documentation methods". It is understandable that hospital staff should choose to pursue a quicker approach to documenting falls if they perceive that the end outcome for themselves and the patient will be the same. The following statement indicates that the respondents viewed documenting the fall in the medical record as being necessary, whereas completing the incident report was optional;

"I would always write it in their chart but not necessarily write up an incident report." r340, nurse.

The effect of the "is staff time available" tertiary determinant was consistent in that, if staff time was not available, respondents indicated that they would be less inclined to complete an incident report. However, the effect of the remaining tertiary determinants was not consistent. For example, the tertiary determinant "Was the fall witnessed" generated directly contrasting effects between individual respondents, for example;

"I am less likely to report a fall if it was witnessed and clearly no injury was sustained." r201, nurse

compared with

"more likely to report if witnessed the fall take place to have all the details." r140, occupational therapist.

Similarly for "Did staff anticipate that the patient would fall";

"More likely (to complete incident report) if patient is confused, elderly, dementia, cognitively impaired or history of falls." r93, nurse.

compared with

"less likely if a near miss during an activity pt considered likely to be at risk ie. Early mobilization or transfers." r142, occupational therapist.

Unexpectedly, the tertiary determinant of "Staff perceive fall caused by patient intrinsic factors " also yielded conflicting responses;

"Some patients just fall because they're older, impulsive and have cognitive deficits" r168, nurse

was provided by one respondent when talking about reasons they do not complete incident reports. In contrast, another respondent found a similar situation to provide greater impetus for recording an incident report and highlighted a link between this tertiary determinant and staff belief that completing incident reports protects against legal liability.

"If the patient is impulsive and has mobilised without the recommended staff assistance I would be more likely to write an incident report to protect staff whom are looking after patient and have recommended to patient not to mobilise independently." r190, nurse.

The inconsistent effect of these tertiary determinants belies inconsistencies in staff perceptions and reasoning for why an incident reports should be recorded for a fall.

The final tertiary determinant was the "Staff perceive the fall could have been prevented" category. This category brought together a number of direct statements similar to;

"If the fall was preventable I would complete an incident report." r127, nurse

Though also displayed an inconsistent effect as evidenced by comments such as

"If it was just poor technique and the patient can and should have known and done better then I probably won't fill one out." r121, nurse

This determinant was considered to be very similar to the "Staff perceive fall caused by patient intrinsic factors" determinant, though conceptually, even if a fall was contributed to by patient intrinsic factors, this does not necessarily mean that staff would perceive that it could have been prevented.

### Environmental/cultural barriers and facilitators

The primary, secondary and tertiary determinants existed within the context of co-related environmental and cultural factors. Environmental/cultural facilitators of "staff belief that completing incident reports improves patient safety" and "staff belief that completing incident reports protects against legal liability" have already been described, though one further example of "staff belief that completing incident reports improves patient safety is worthy of specific mention.

"It is not possible to quickly refer to incident reports previously submitted in the charts so that if there is more than one, we are not able to do a quick check to see if there is a common cause for the fall." r21, nurse.

In some of the participating hospitals, incident reports were not filed within the medical progress notes of patients, so that staff were unable to review previous reports to find common elements that may be causing ongoing falls for individual patients. Thus even though the fall may have had preventable elements consistent with previous falls, the restricted access to this data by clinicians treating a patient serves as a disincentive for reporting.

The environmental/cultural barriers of "poor user friendliness of computerized reporting system" and "presence of alternative documentation methods" have also already been described. Examples of comments depicting the remaining barriers include;

i) the perception that the act of completing an incident made staff feel personally responsible for the fall:

"Less likely to complete because ... (completing an incident report) makes you feel responsible for the patient falling." r208, nurse

ii) poor access to computers to use the computerized incident reporting system:

"Less likely to complete because decreased access to computers" r159, occupational therapist

iii) Non-reporting by role models

"Role models – if nursing medical staff are compliant to reporting falls, I'm more likely to report falls" r116, physiotherapist.

iv) Absence of a definition of a fall

"A standardized definition of fall would make it easier to identify fall and report accordingly" r116, physiotherapist

v) Perceived blame from others

"Less (likely) because of perceived blame to person completing incident report." r212, nurse

iv) Absence of training for computerized reporting systems

"Training for XXXX (computer program) would be helpful to decrease time to complete the forms" r63, nurse

Analysis of results by ward and hospital revealed clustering of comments (24 of 51) in the "poor user friendliness of computerized reporting system" category originating from seven wards within one participating hospital. Clustering of responses for this particular category may have been symptomatic of local processes involved in the implementation of this program as the program itself was the same across the state. No further clustering of comments by ward, hospital or professional discipline was evident.

## Discussion

This research has identified a complex hierarchy of determinants that can impact upon recording of hospital falls on incident reports that exist within the context of a range of environmental/cultural factors that can also impede incident reporting. Previous authors have suggested that falls may be "habitually" reported,[7,8] and have implied that they have collated datasets without missing falls data.[11,12] Yet the present research brings such assumptions into question given the numerous comments which imply that staff members do not adopt the principle of reporting all falls on incident reports. This is supported by other previous work that has questioned the "completeness" of in-hospital falls incident reporting.[13,14]

Previous research has sought to identify factors that impact upon incident reporting (in general) in hospitals.[7,8,15] This research is the first to focus specifically on the process of reporting falls and has identified a distinctive characteristic impacting of the reporting of in-hospital falls. Unlike some other incident types (eg. medication error) there was a perception falls may be caused by factors intrinsic to the patient rather than as solely being the fault of the staff member. Given the evidence identified that indicates hospital staff complete incident reports (in part) to protect themselves against legal liability, it was intriguing to observe the divergent effects this had on the likelihood of completing an incident report. For some, belief that patients contributed to the fall reduced the likelihood of completing an incident report. It is possible that these staff felt that they were less likely to face disciplinary action or legal ramifications if they were not at fault, and thus were not motivated to complete an incident report. However for others, perceiving that the patient contributed to the fall increased their likelihood of reporting. These staff may feel the risk of facing disciplinary action or legal ramifications does not depend on their own assessment of fault, and complete an incident report to generate evidence to safeguard themselves against this. Highlighting additional risk staff face in having legal action succeed if brought against them if an incident report is not completed may help enforce the latter attitude amongst staff. This could take the form of case studies provided during staff training, which could also highlight cases where staff have been found to be at fault even though they thought they were not.

Other strategies aimed at improving the consistency and completeness of recording falls on incident reports emerge from this research. A small number of staff provided comments indicating that they perceived that completing incident reports improves patient safety, and that this facilitated completion of incident reports. Increased levels of incident reporting could therefore potentially be attained if all staff held the belief that incident reporting improves patient safety. To achieve this, case-studies could be provided to staff demonstrating how incident reports have been used to prompt falls prevention strategies for individual patients, and examples could also be provided of how incident reports have been used to develop and evaluate "system-level" interventions. Similarly, post-fall reviews could be undertaken as a part of routine care where staff caring for a repeat faller would review the information from previous fall-related incident reports to identify commonalities between these falls that could be the focus of intervention for that patient. To achieve this however, hospitals would need to ensure that all treating hospital staff had ready access to all the fall-related incident reports for their patients, a practice not universally in place.

Other recommendations arising from this research are; that a standardized definition of a fall be applied and that staff be trained in how to apply this definition to a range of clinical scenarios that they might encounter, that alternatives to computer-based data entry (such as a telephone hotline) be explored particularly where computer literacy or access to computers cannot be assured, that training as to how to complete an incident report be made mandatory for all hospital staff, and that senior hospital staff be charged with the responsibility of being role models for recording every fall on an incident report and spreading this message amongst their staff.

### Study limitations

This study may have been unable to fully capture the range and depth of contextual factors impacting upon recording of in-hospital falls on incident reports. The selection of wards for participation in this study was on the basis of high recorded falls rates (from local data). It is possible that there were wards with high "real" falls rates but with low recording rates that would not have been selected. The written response format used in this research did not allow investigators to return to respondents to clarify their responses and to further probe to explore deeper issues relating to those stated. Similarly, the prompt employed may have biased respondents towards providing more information regarding contextual factors that were barriers to reporting rather than facilitators. Future research could employ alternate data collection approaches (in-depth interviews, focus groups) to examine whether the conclusions reached in the present study are justified. The present study also had a high rate of surveys returned where responses to the question being investigated in the present project were not provided. This may have generated a selection bias, whereby the people who chose not to respond to this section of the survey may have held a different perspective on recording falls on incident reports than that captured within the present study. This risk was apparent to investigators at project outset, however the investigators felt that the potential risk of selection bias in the context of the present research question was less of a concern than ensuring that the design promoted the provision of open and honest answers. If face-to-face interviews were able to be administered, it is possible that these non-responders may have discussed issues or highlighted relationships between categories that were not identified in the present study.

## Conclusion

In-hospital falls continue to be a common and concerning adverse event amongst hospital inpatients. Conduct of valid research and clinical monitoring of this area is contingent upon accurate and complete recording of in-hospital falls on incident reports where hospital incident reporting systems are the source of falls data. This research has established a contextual framework through which the propensity for a hospital staff member to complete and incident report for a fall can be understood. Understanding this framework and the factors motivating its structure has allowed for the development of several recommendations aimed at improving completeness and consistency in recording of falls on incident reports.

## Competing interests

The authors declare that they have no competing interests.

## Authors' contributions

TH was responsible for study conception, design, analysis, interpretation and writing of manuscript. PC was responsible for analysis, interpretation and assisted in writing of manuscript. JF assisted in study conception, design, analysis, interpretation and writing of manuscript. PV, LG assisted in study conception, design and writing of manuscript.

## Pre-publication history

The pre-publication history for this paper can be accessed here:


